# The critical role of Rap1-GAPs Rasa3 and Sipa1 in T cells for pulmonary transit and egress from the lymph nodes

**DOI:** 10.3389/fimmu.2023.1234747

**Published:** 2023-07-20

**Authors:** Shunsuke Horitani, Yoshihiro Ueda, Yuji Kamioka, Naoyuki Kondo, Yoshiki Ikeda, Makoto Naganuma, Tatsuo Kinashi

**Affiliations:** ^1^ The Department of Molecular Genetics, Institute of Biomedical Science, Kansai Medical University, Hirakata, Japan; ^2^ Division of Gastroenterology and Hepatology, the Third Department of Internal Medicine, Kansai Medical University, Hirakata, Japan

**Keywords:** Rap-GAP, T cell trafficking, integrin, lung, Rap1, LFA1, T-cell recirculation, egress

## Abstract

Rap1-GTPase activates integrins and plays an indispensable role in lymphocyte trafficking, but the importance of Rap1 inactivation in this process remains unknown. Here we identified the Rap1-inactivating proteins Rasa3 and Sipa1 as critical regulators of lymphocyte trafficking. The loss of Rasa3 and Sipa1 in T cells induced spontaneous Rap1 activation and adhesion. As a consequence, T cells deficient in Rasa3 and Sipa1 were trapped in the lung due to firm attachment to capillary beds, while administration of LFA1 antibodies or loss of talin1 or Rap1 rescued lung sequestration. Unexpectedly, mutant T cells exhibited normal extravasation into lymph nodes, fast interstitial migration, even greater chemotactic responses to chemokines and sphingosine-1-phosphate, and entrance into lymphatic sinuses but severely delayed exit: mutant T cells retained high motility in lymphatic sinuses and frequently returned to the lymph node parenchyma, resulting in defective egress. These results reveal the critical trafficking processes that require Rap1 inactivation.

## Introduction

Naïve T cells recirculate between the blood and secondary lymph nodes (LNs) for immune surveillance. During recirculation, T cells enter LNs *via* high endothelial venules (HEVs) expressing peripheral lymph node addressin (PNAd) and integrin ligands intercellular adhesion molecule (ICAM)1, ICAM2, and vascular cell adhesion molecule 1 (VCAM1). This process is mediated by a multi-step adhesion cascade consisting of L-selection-dependent rolling followed by chemokine-induced integrin-mediated arrest. LFA1 and α4 integrins are major integrins that mediate arrest and have an additional role in supporting rolling (1, 2). After firm attachment to HEVs, T cells extravasate into the LN parenchyma and migrate into the paracortical T-cell zone, where they randomly move along networks of stromal cells and dendritic cells *via* CCR7-mediated actin dynamics and LFA1-mediated friction (3–5). Eventually, T cells enter LYVE1^+^ lymphatic sinuses by chemotactic responses to sphingosine-1-phosphate (S1P), leading to egress from LNs (6, 7) and return to circulation.

The small GTPase Rap1 plays vital roles in integrin-mediated lymphocyte adhesion and polarity upon antigenic or chemokine stimulation (8, 9). Rap1 recruits the integrin adaptors talin1 and kindlin-3 to bind the β cytoplasmic tail of integrins, thereby cause integrin conformation and affinity changes (10–12). Integrin activation by Rap1 and talin1 is indispensable for T-cell arrest on HEVs, and the loss of Rap1 or talin1 causes severe lymphocyte homing defects (13–16).

Rap1 is tightly regulated by the activation of guanine exchange factors (GEFs) and the inactivation of GTPase-activating proteins (GAPs), and it cycles between an inactive GDP-bound form and an active GTP-bound form. RasGRP2 (Cal-DAG-GEFI) and C3G (RapGEFI) regulate Rap1 activation in leukocytes and platelets (17–22). Sipa1 is a Rap-specific GAP that is abundantly expressed in hematopoietic cells, including lymphocytes (23). Overexpression of Sipa1 was shown to inhibit Rap1-induced cell adhesion, cell polarity (9, 24), and thymocyte development (25). Sipa1-deficient mice were reported to develop late-onset autoimmunity or myeloproliferative diseases (26, 27). Rasa3 is a member of the Ras-GAP1 family with dual GAP activity for Rap1 and Ras (28). While inactivation of Rasa3 was embryonic lethal, *scat* mice carrying a missense mutation of Rasa3 exhibited severe anemia and thrombocytopenia (29). Bone-marrow chimeras reconstituted with Rasa3-deficient fetal liver or hypomorphic Rasa3 mutations demonstrated linkage of Rap1 and the integrin αIIbβ3, leading to platelet activation and severe thrombopenia (30, 31). T-cell–specific deletion of Rasa3 did not appear to affect T-cell homeostasis in the thymus or periphery but did impair Th17 production in experimental autoimmune encephalomyelitis model mice (32). A recent study reported that the lack of Rasa3 in T cells increased T-cell receptor (TCR)–triggered binding to soluble ICAM1 and impaired T-cell entry into and egress from peripheral LNs (33). However, the precise mechanisms by which Rap-GAPs regulate T-cell trafficking have not been fully elucidated.

Here we showed that the absence of Rasa3 in T cells caused severe decreases of T cells in the blood and peripheral LNs, and this was aggravated by Sipa1 deficiency. We found that Rasa3 and Sipa1 double knockout (DKO) T cells were entrapped in ICAM^hi^ lung capillary beds *via* LFA1, and this entrapment was rescued by the lack of Rap1 and talin1. While LN entry and intranodal migration of DKO T cells were comparable to those of wild-type (WT) T cells, they exhibited severely defective egress from LNs. Two-photon imaging revealed that after entry into lymphatic vessels, the mutant T cells maintained high motility and frequently migrated back to the LN parenchyma, a process that was partially dependent on ICAM1 expression (34). Our study clarifies the critical processes of T-cell recirculation that require Rap1 inactivation by Rasa3 and Sipa1.

## Materials and methods

### Mice

*Rap1a^flox/flox^*, *Rap1b^flox/flox^*, *Rasa3^flox/flox^*, and *Tln1^flox/flox^* mice were described previously (31, 35, 36). *Sipa1*-knockout mice were generated in our laboratory using Crispr/Cas9 technology with a guide RNA targeting exon 7 of the mouse *sipa1* gene (TACACGCCTAATAACCAG). *Cd4-cre* transgenic mice for T-cell–specific deletion were obtained from Taconic. These strains were intercrossed to generate double and triple knockout mice. Littermates or age-matched adult *Rasa3^flox/flox^* mice (6–24 weeks old) were used as WT controls. C57BL/6 mice were obtained from CLEA Japan Inc. All mice were maintained under specific pathogen–free conditions in the animal facility at Kansai Medical University. All animal experiments were performed in accordance with protocols approved by the Animal Care and Use Committee of Kansai Medical University (approval no. 21-097).

### Antibodies and reagents

FITC-, PE-, PerCP-Cy5.5–, Alexa Fluor 647–, APC-, eFluor 450–, and eFluor 660–conjugated antibodies specific for AnnexinV, B220 (RA3-6B2), CD3 (145-2C11), CD4 (GK1.5, RM4-5), CD8 (53-6.7), CD31 (390), CD44 (IM7), CD54 (YN1/1.7.4), CD62L (Mel-14), CD69 (H1.2F3), CD106 (429), LYVE1 (ALY7), and PNAd (MECA-79) were purchased from BioLegend or Invitrogen. Anti-CCL21 and PE-labeled anti-S1P1 (713412) were purchased from R&D Systems. FITC-labelled TCRβ (H57-597) was purchased from BD Biosciences. Alexa Fluor 488– or 633–conjugated goat anti-mouse, anti-rat, or anti-human IgG (1–2 μg/mL) was purchased from Jackson ImmunoResearch Laboratories. Anti-LFA1 (KBA) and anti-CD62L (MEL) for neutralization was prepared from our laboratory. CFSE (5- or 6-(N-succinimidyloxycarbonyl)-fluorescein 3′,6′-diacetate), CMTMR ((5-(and 6)-(((4-chloromethyl)benzoyl)amino) tetramethylrhodamine), and CMF2HC (4-chloromethyl-6,8-difluoro-7-hydroxycoumarin) dye for cell labeling were obtained from Invitrogen. Recombinant CCL21 was purchased from R&D Systems and S1P and FTY720 were obtained from Cayman Chemicals.

### Flow cytometry

Cells (1−2 × 10^6^) were stained with fluorescent antibodies in culture medium (Iscove′s Modified Dulbecco′s medium supplemented with 2-mercaptoethanol and 4% fetal calf serum (4% FCS IMDM) and analyzed with an Attune NxT Flow Cytometer (Thermo Fisher) or a FACS Canto II flow cytometer (BD Bioscience). Acquired data were analyzed using FlowJo software.

### Detection of Rap1-GTP by pull-down assays

Purified T cells (0.5–2 × 10^7^) in RPMI were lysed with one volume of 2 x lysis buffer (2% Triton X, 0.2 M Tris-HCl pH 7.5, 0.3 M NaCl, 30 mM MgCl_2_, aprotinin, PMSF) and subjected to pull-down assays using Glutathione Sepharose 4B (GE Healthcare) conjugated with GST-RalGDS-RBD for Rap1. Immunoblotting was performed by using antibodies specific for Rap1 (36)

### Detachment assay

Detachment assay was performed as described previously (2). Anti-human IgG Fc specific antibodies (100 μg/mL, Rockland Immunochemicals) were coated overnight on a plastic dish and washed with PBS. Recombinant mouse ICAM1-Fc (1 μg/mL) was loaded on the disk and incubated for >2 h at room temperature. Then, the disk was placed in a FCS2 flow chamber (Bioptechs). Purified T cells (2 × 10^6^/mL) in a warm medium (IMDM containing 1% FCS) were loaded into the chamber, and bright-field images were recorded every 5 s with a 4× objective lens and CCD camera (HAMAMATSU C2741). Cells were settled on ICAM1 for 5 minutes, shear stress was applied for 1 min at 2 dyn/cm2, with a stepwise increment of 1 dyn/cm2 every minute to 5 dyn/cm^2^, using a programmed syringe pump (FP-1000, MELQUEST). The attached cells were counted by using Metamorph (Molecular Devices). Adhesion efficiencies were calculated as the percentages of attached cell numbers relative to input cell numbers.

### Immunohistochemistry

Mouse tissues were embedded in OCT compound (Sakura Finetek) and frozen at -80 °C. Frozen sections (10-μm thick) were cut by a cryostat (Leica Biosystems) and fixed with cold acetone or 4% paraformaldehyde (PFA) in PBS. Fixed sections were stained for 2 hours with antibodies specific for ICAM1, ICAM2, and VCAM1 diluted in blocking solution. The samples were mounted, and the images were acquired using a FV3000 confocal microscope (Olympus). Some tissues were fixed with 4% PFA and cut with a vibratome. The vibratome sections were permeabilized with PBS containing 0.5% TritonX100 for 10 minutes and were blocked with PBS containing 0.3% TritonX100, 1% BSA, and 2% mouse/rat serum for 2 hours. The sections were stained for 1 day in blocking solution with a combination of antibodies specific for CD3, CD31, VCAM1, LYVE1, or CCL21. Images of stained sections were acquired by confocal microscopy (FV3000, Olympus).

### Homing assay

T cells were differentially labeled at 37°C for 20 min with CMTMR (1 μM), CFSE (1 μM), or CMF_2_HC (25 μM) in RPMI 1640. Equal numbers of labeled WT and mutant T cells (5−10x10^6^ each) in PBS were intravenously injected into each recipient. In some experiments, T cells were pretreated with 100 μg/ml control antibody or anti-LFA1 antibody at 37°C for 20 minutes and subjected to adoptive transfer. Surface LNs, spleen, and blood were collected from each recipient at 1 hour or 24 hours. The samples were analyzed by flow cytometry to detect CMTMR- or CFSE-labeled donor cells. The relative homing efficiency of KO T cells in each tissue was assessed as the ratio of the percentage of KO T cells to that of WT cells in tissues after normalization with the input ratio in the blood.

### LN egress assay

Labeled KO and WT T cells were intravenously injected into recipient mice. Twenty-four hours after injection, 200 µg of anti-L-selectin antibody (MEL14, 200 μg) was intravenously injected into the recipient mice. In some mice, 50 μg of FTY720 was intraperitoneally administered twice in addition to anti-CD62L antibody. Eight or 24 hours after MEL14 antibody injection, CMTMR- or CFSE-labeled cells in LNs were analyzed by flow cytometry. The relative egress efficiency of KO T cells was assessed as the ratio of the percentage of KO T cells to that of WT cells in tissues after normalization with the input ratio in the blood. In another experiment, frozen sections of isolated LNs were stained with fluorescently labeled anti-LYVE1 antibody to visualize lymphatic sinuses. The density of labeled WT and KO T cells in the LN parenchyma was measured by ImageJ software to calculate the ratio of KO T-cell density to WT T-cell density.

### Live LN imaging

WT and DKO T cells labeled with CFSE and CMTMR were adoptively transferred into recipient mice. Twenty-four hours after transfer, a footpad of each recipient mouse was injected with eFluor 450–conjugated anti-LYVE1 to visualize lymphatic sinuses. LNs were harvested 2 hours after injection and subjected to imaging with a 2–photon laser scanning microscope. Two-photon imaging of LN explants was performed as described previously (3). In brief, LNs isolated from recipient mice were glued onto plastic coverslips (Thermo Fisher Scientific) with Vetbond (3M) and then enclosed with low-gelling agarose. The samples were placed in a heated chamber (Warner Instruments) and continuously perfused with RPMI 1640 medium bubbled with 95% O_2_/5% CO_2_ at 36.0–37.0 °C. Time-lapse images were acquired using a FV1000 upright microscope (Olympus) with a Ti:sapphire laser (MaiTai HP DeepSee-OL, Spectra Physics) tuned to 825−850 nm and a X25/1.05 NA objective (XLPLN25XWMP, Olympus). Emitted light was passed through 420−460 nm, 495−540 nm and 575−630 bandpass filters to a photomultiplier for detection of eFlour 450, CFSE, and CMTMR fluorescence. Acquisition was controlled with FV10-ASW software (Olympus), and Z stacks were typically acquired every 15–20 s, with Z steps spaced 2.5 μm apart. Time-lapse projection images were generated from two continuous z planes containing LYVE1^+^ lymphatic sinuses. Then, the manual mode of Trackmate plug-in in ImageJ Fiji software were used for tracking cells in the images. Alternatively, Imartis software (Bitplane) with the spot option (assuming a 5-μm sphere) was used for tracking cell positions over time in 3 dimensions. To measure cell localization relative to the outside, inside, and border of LYVE1^+^ lymphatic sinuses, the LYVE1^+^ area was rendered with the surface option and the distance of transferred T cells from the surface was calculated using the surface distance option of the Imaris software. Obtained data were analyzed using Microsoft Excel software.

### Measurement of lymphatic sinus entry

T cells were labeled with fluorescent dye and adoptively transferred into recipient mice. MEL14 antibodies were injected 1 hour after adoptive transfer to inhibit further entry. Four hours after antibody injection, surface LNs were harvested and fixed with 1% PFA in PBS. The LNs were cut into 300-μm-thick sections with a vibratome and stained with anti-LYVE1 and anti-PNAd antibodies. The Z-stack (2 μm) images were acquired using a FV3000 microscope with a 10x objective and Z steps spaced 2 μm apart. The cell numbers of the labelled cells in HEVs, the LN parenchyma and lymphatic sinuses were counted from 2 microscopic fields per a LN slice.

### Chemotaxis assay

A chemotaxis assay was performed using a Transwell system (Corning Costar) with 5-μm pore size. To measure migration to CCL21, 1 × 10^6^ freshly isolated T cells in 100 μl of 1% FCS RPMI 1640 were loaded onto the upper chamber and were allowed to migrate to CCL21 at the indicated concentrations in the lower chamber for 1 hour (600 μl of 1% FCS RPMI 1640) at 37°C and 5% CO_2_. In some experiments, the upper chamber was coated with 1, 10, or 100 μg/ml of ICAM1-Fc overnight before use. To examine migration toward S1P, isolated T cells or thymocytes were incubated for 3 hours in RPMI 1640 medium containing 0.1% fat-free BSA. Then T cells (1 × 10^6^) or thymocytes (2 × 10^6^) were loaded onto the upper chamber and allowed to migrate to S1P (3−300 nM). To assess the migration of CD62L^hi^CD4 SP thymocytes, cells migrated into the lower chamber were stained with fluorescently labeled antibodies specific for CD4, CD8, and CD62L and analyzed by flow cytometry.

## Results

### Spontaneous Rap1 activation and adhesion of T cells deficient in Rasa3 and Sipa1

**Figure 1:**
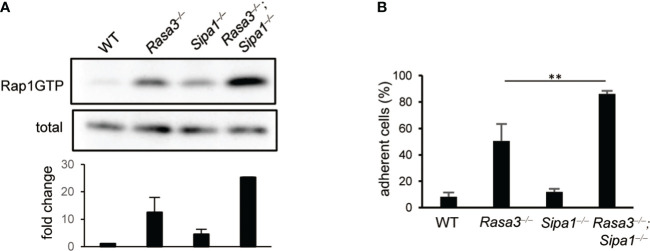
Rasa3- and Sipa1-deficient T cells exhibited spontaneous adhesion and an enhanced migration response to CCL21. **(A)** Rap1 activation of of Rasa3-KO (*Rasa3^–/–^*), Sipa1-KO (*Sipa1^–/–^*) and DKO (*Rasa3^–/–^Sipa1^–/–^*) T cells in the absence of chemokines. Rap1 activation was measured by a pull-down assay using GST-RalGDS-RBD. The lower bar graph indicates the average amount ( ± SD) of Rap1-GTP normalized to total (n = 2). **(B)** Spontaneous adhesion of Rasa3-KO and DKO T cells. The adhesive responses to ICAM1 (capture antibody (30 µg/ml) + mouse ICAM1-Fc 1 µg/ml) of Rasa3-KO, Sipa1-KO and DKO T cells were measured by a detachment assay (n = 3). The bar graph shows the average number of adherent cells at 3 dyn under sequential shear flow (see Methods). The statistical significance of the above data was calculated by Student’s t-test. ^**^ *P* <.01.

To evaluate the contribution of Rasa3 and Sipa1 to Rap1 suppression in naïve T cells, we measured the amount of Rap1-GTP in T cells from T-cell–specific *Rasa3* knockout (KO) mice and *Sipa1*-KO mice (*Cd4-cre Rasa*3*^fl/fl^* and *Sipa1* ^–/–^, respectively). Compared to WT T cells (*Rasa3^fl/fl^*), the basal amounts of Rap1-GTP were increased approximately 12-fold and 4-fold in *Rasa3*-KO and *Sipa1*-KO T cells, respectively (**Figure 1A**). The amount of Rap1-GTP was further increased by 25-fold in DKO T cells (**Figure 1A**). Thus, both Rasa3 and Sipa1 contributed to the suppression of Rap1 in T cells. Constitutively active Rap1 induces adhesive responses to integrin ligands in the absence of stimulation (9). The loss of Rasa3 also consistently induced spontaneous adhesion of T cells to ICAM1 (**Figure 1B**). The absence of both Rasa3 and Sipa1 further increased this adhesive response, indicating their major role in maintaining LFA1 in a low-affinity state.

### Decreased numbers of peripheral T cells in DKO mice

**Figure 2:**
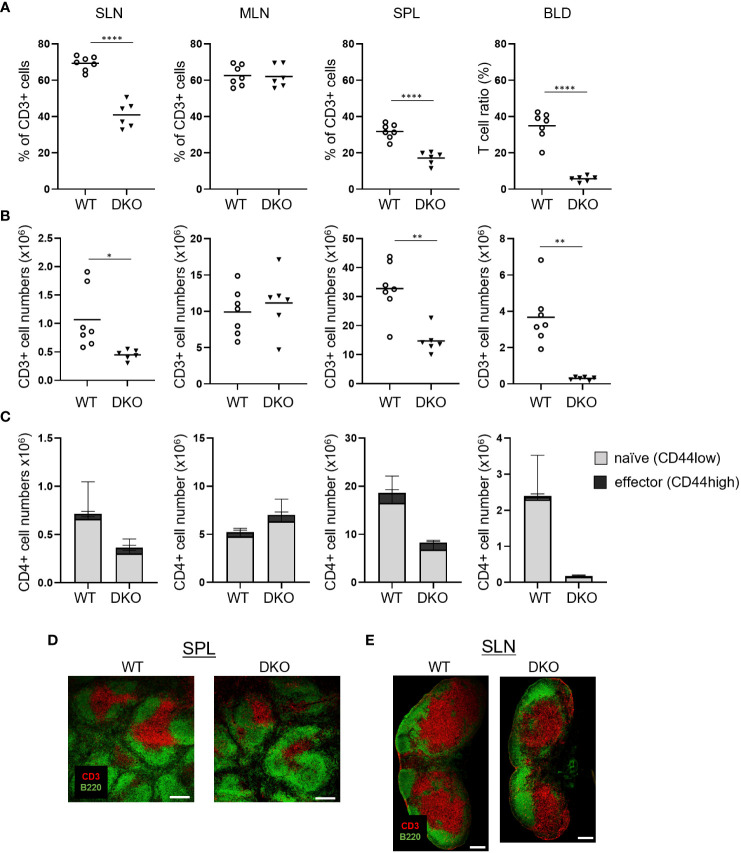
T-cell lymphopenia in Rasa3- and Sipa1-deficient mice. **(A)** The percentage of CD3^+^ T cells in superficial LNs (SLN), mesenteric LNs (MLN), spleen (SPL), and blood (BLD) from WT (*Rasa3* ^fl/fl^) and DKO (*Cd4-creRasa3^f/f^ Sipa1^–/–^*) mice, measured by flow cytometry. Bars indicate mean ± SD. **(B)** The numbers of CD3^+^ T cells in SLNs, MLNs, SPL, and BLD from the mice in **(A)**. **(C)** The numbers of CD44^low^ naïve (grey) and CD44^high^ effector (black) CD4^+^ T cells in SLNs, MLNs, SPL, and BLD of the above mice. Bars indicate mean ± SD. **(D)** Decreased area of splenic T-cell zones in DKO mice. Confocal images show CD3^+^ T-cell zones (red) and B220^+^ B-cell follicles (green) in spleens of WT and DKO mice. Scale bar, 200 μm. **(E)** Smaller T-cell zones in the superficial LNs of DKO mice. Confocal images show T-cell zones (red) and B-cell follicles (green) of LNs from WT and DKO mice. Scale bar, 200 μm. The statistical significance of the above data was calculated by Student’s t-test. ^*^ *P* <.05, ^**^ *P* <.01, ^****^ *P* <.0001.

We examined the impact of the loss of Rasa3 and Sipa1 in T cells *in vivo*. Compared to WT mice, *Rasa3*-KO mice exhibited decreases in both the percentages and numbers of CD3^+^ T cells in superficial LNs (SLNs) and blood, while T-cell numbers were normal in *Sipa1*-KO mice (**Figures S1A, B**), as previously reported (26, 33). The absence of Rasa3 and Sipa1 decreased the numbers of CD3^+^ T cells in SLNs and to a greater extent in the spleen and blood (**Figures 2A, B**; **S1A, B**). In contrast to these results, the numbers of effector CD44^high^ CD4^+^ T cells were relatively unchanged (**Figure 2C**). The numbers of B220^+^ B cells in the blood and lymphoid tissues were normal in mutant mice, except for an unexplained significant decrease in the number of B cells in SLNs of T-cell–specific Rasa3-KO mice **(** **Figure S1C**). Immunostaining of the spleen and LNs of DKO mice showed smaller and less dense T-cell zones compared to those of WT mice (**Figures 2D, E**). Thus, a loss of Rasa3 alone caused T-cell lymphopenia in the periphery, which was exacerbated by the additional loss of Sipa1 in correlation with Rap1 activation levels (**Figure 1**).

To explore the underlying mechanisms of peripheral T-cell lymphopenia in DKO mice, we examined thymocyte subsets (**Figure S2**). The ratios and cell numbers of CD4/CD8 double-positive (DP) cells and CD4 or CD8 single-positive (SP) cells were unchanged in DKO mice, suggesting normal thymocyte development (**Figures S2A, B**). An increased number of CD69*^–^* CD62L^+^ SP cells is a common feature of defective thymocyte egress (6, 37). However, the ratios and numbers of immature CD69^+^CD62L*^–^* and mature CD69*^–^* CD62L^+^ CD4 SP cells were unchanged in DKO mice compared to those of WT mice (**Figures S2C, D**). Thymocyte egress depends on chemotaxis to sphingosine 1-phosphate (S1P) (6). Mature CD4 SP cells from DKO mice migrated to S1P as efficiently as WT T cells (**Figure S2E**). Collectively, these data suggest that thymocyte development and egress occurs normally in DKO mice.

### Reduced homing of DKO T cells to lymphoid tissues

**Figure 3:**
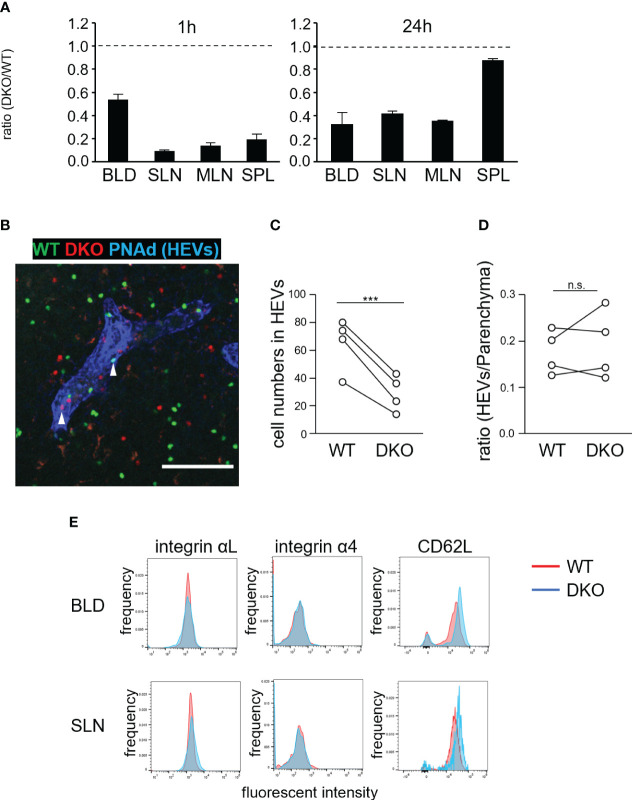
Rasa3- and Sipa1-deficient T cells exhibit defective homing to LNs but normal transmigration *via* HEVs. **(A)** Inefficient homing of DKO T cells to lymphoid tissues. Equal numbers of WT and DKO T cells were adoptively transferred to WT mice. At 1 hour and 24 hours, the transferred T cells present in blood (BLD), superficial LNs (SLN), mesenteric LNs (MLN), and spleen (SPL) were measured by flow cytometry. The ratio of DKO T cells to WT T cells is shown (n = 3). **(B)** WT (green) and DKO cells (1:2.6*–*2.7 ratio) were adoptively transferred to WT mice. One hour after transfer, LNs were harvested, fixed, cut with a vibratome, and stained with anti-PNAd and anti-LYVE1 to visualize HEVs and lymphatic sinuses, respectively. A representative projection image of WT (green) and DKO T cells (red) and HEVs (blue) is shown. Arrows indicate transferred T cells in HEVs. **(C)** The numbers of transferred WT and DKO T cells in HEVs within a microscopic field of LN slices are shown. The numbers are normalized to the ratio of input cells (4 LNs from 2 mice). **(D)** The efficiency of transmigration of DKO T cells from the HEVs to the LN parenchyma in the above LN slices. The efficiency was calculated as the ratio of the cell number in the HEVs to that in the parenchyma of the cut LN slice in **(C)**. **(E)** Expression of integrin αL, integrin α4, and CD62L on WT (red) and DKO T cells (blue). The statistical significance of the above data was calculated by Student’s t-test. ***P<.001, n.s: not significant.

We next examined the homing abilities of DKO T cells. Differentially labeled WT and DKO T cells were adoptively transferred into WT mice. One hour later, the ratios of DKO T cells to WT T cells in SLNs, mesenteric LNs (MLNs), spleen, and blood were determined (**Figure 3A**). The ratios of DKO T cells to WT T cells in LNs and spleen decreased to 0.1–0.2. In addition, the ratio of DKO T cells in the blood decreased to approximately 0.5 (**Figure 3A**). After 24 hours, the ratios of DKO T cells to WT cells increased to 0.3–0.4 in LNs and to 0.9 in the spleen (**Figure 3A**). Thus, DKO T cells exhibited defective homing to the lymph nodes and delayed homing to the spleen.

To clarify defective T-cell homing, we examined the distribution of WT and DKO T cells in LNs 1 hour after adoptive transfer (**Figures 3B–D**). HEVs were identified by staining with a fluorescently labeled anti-PNAd antibody. The number of DKO T cells in HEV lumens was approximately half that of WT T cells (**Figure 3C**). By contrast, the ratio of the number of cells in the LN parenchyma to that in HEVs was comparable for DKO T and WT T cells **(** **Figure 3D** **)**, indicating that DKO T cells extravasated to the LN parenchyma as efficiently as WT T cells. DKO T cells normally expressed αL and α4 integrins with slightly higher levels of CD62L compared to WT T cells in the blood and SLNs **(** **Figure 3E** **)**.

### DKO T cells were entrapped in the lungs by activated LFA1

**Figure 4:**
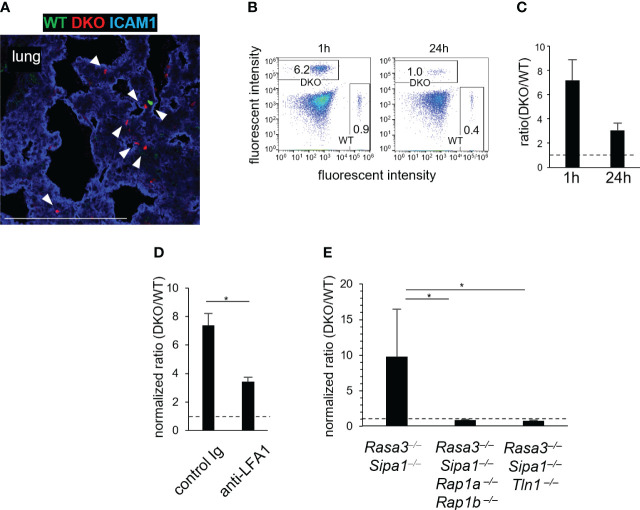
Rasa3- and Sipa1-deficient T cells are entrapped in the lungs in a manner dependent on LFA1, Rap1, and talin1. **(A)** WT T cells are entrapped in the lungs. A confocal image is shown of WT T cells (green), DKO T cells (red), and ICAM1 (blue) in the lungs of recipient mice 1 hour after the adoptive transfer. Arrow heads showed the transferred T cells. **(B)** Representative fluorescence-activated cell sorting profiles of transferred WT (CFSE) and DKO (CMTMR) T cells in the lungs 1 hour and 24 hours after adoptive transfer. **(C)** The ratio of DKO T cells to WT T cells in the lungs 1 hour and 24 hours after adoptive transfer. **(D)** Entrapment of DKO T cells in the lungs depended on LFA1. DKO T cells were pretreated with control antibody or anti-LFA1 antibody (200 μg, 30 min). The efficiency of homing to the lungs was measured 1 hour after adoptive transfer. The ratio of DKO T cells to WT T cells in the lungs was corrected to that in the blood. **(E)** The entrapment of DKO T cells in the lungs depended on Rap1 and Talin1. The efficiency of homing to the lungs was measured using T cells from *Cd4-cre Rasa3* ^fl/fl^ *Sipa1^–/–^ Rap1a* ^fl/fl^ *Rap1b* ^fl/fl^ mice (*Rasa3^–/–^Sipa1^–/–^Rap1a^–/–^Rap1b^–/–^*) and *Cd4-cre Rasa3* ^fl/fl^ *Sipa1^–/–^ Tln1* ^fl/fl^ mice (*Rasa3^–/–^Sipa1^–/–^Tln1^–/–^*). The ratio in the lungs was corrected as in **(D)**. The statistical significance of the above data was calculated by Student’s t-test. ^*^ *P* <.05.

Since DKO T cells showed spontaneous adhesion to ICAM1 (**Figure 1B**), we speculated that transferred DKO T cells were trapped in non-lymphoid tissues, causing a decrease in the number of T cells in the blood and hence in LNs as well. To investigate this possibility, we adoptively transferred WT and DKO T cells to WT recipients and examined their distribution in various tissues. DKO T cells were trapped in the lungs (**Figure 4A**; **S3A**). The lungs exhibited the highest expression of ICAM1 among tissues examined **(** **Figures S3A, B**). ICAM2 was also expressed ubiquitously in the lungs, but VCAM1 expression was restricted to large vessel walls (**Figure S3B**), as previously reported (38). The transferred DKO T cells were scattered throughout the lungs and localized with ICAM1^+^ cells (**Figure 4A**; **S3A**). Flow cytometric analysis showed there were approximately 7-fold more DKO T cells than WT T cells in the lungs at 1 h after transfer, and 3-fold more at 24 hours (**Figures 4B, C**).

To clarify the roles of the LFA1 and integrin activators Rap1 and talin1 in lung entrapment, we adoptively transferred WT and DKO T cells with anti-LFA1 antibody and measured their accumulation in the lungs (**Figure 4D**). In this experiment, the DKO/WT T-cell ratios in the lungs were assessed after normalization with those in the peripheral blood, because the inhibition of integrins or integrin adaptors has been shown to increase T cell numbers in the blood due to lymphocyte homing defects (13, 16, 39). The anti-LFA1 treatment decreased the ratio in the lungs by half, indicating that the entrapment of DKO T cells involved LFA1 (**Figure 4D**). To investigate the role of Rap1 and talin1 in this process, we measured the entrapment of transferred DKO T cells lacking Rap1 (*CD4-cre Rasa3* ^fl/fl^ *Sipa1* ^−/−^ *Rap1a* ^fl/fl^ *Rap1b* ^fl/fl^ mice) or talin1 (*CD4-cre Rasa3* ^fl/fl^ *Sipa1* ^−/−^ *Tln1* ^fl/fl^ mice) (**Figure 4E**). The accumulation of DKO T cells in the lungs was abolished in the absence of Rap1 or talin1 (**Figure 4E**). Collectively, these data indicate that transferred DKO T cells were entrapped by attachment to the pulmonary vascular bed by integrins, including LFA1, in a process that depended on Rap1 and talin1.

### Accumulation of T cells in CCL21^+^ lymphatic vessels of the lungs in Rasa3- and Sipa1-deficient mice

**Figure 5:**
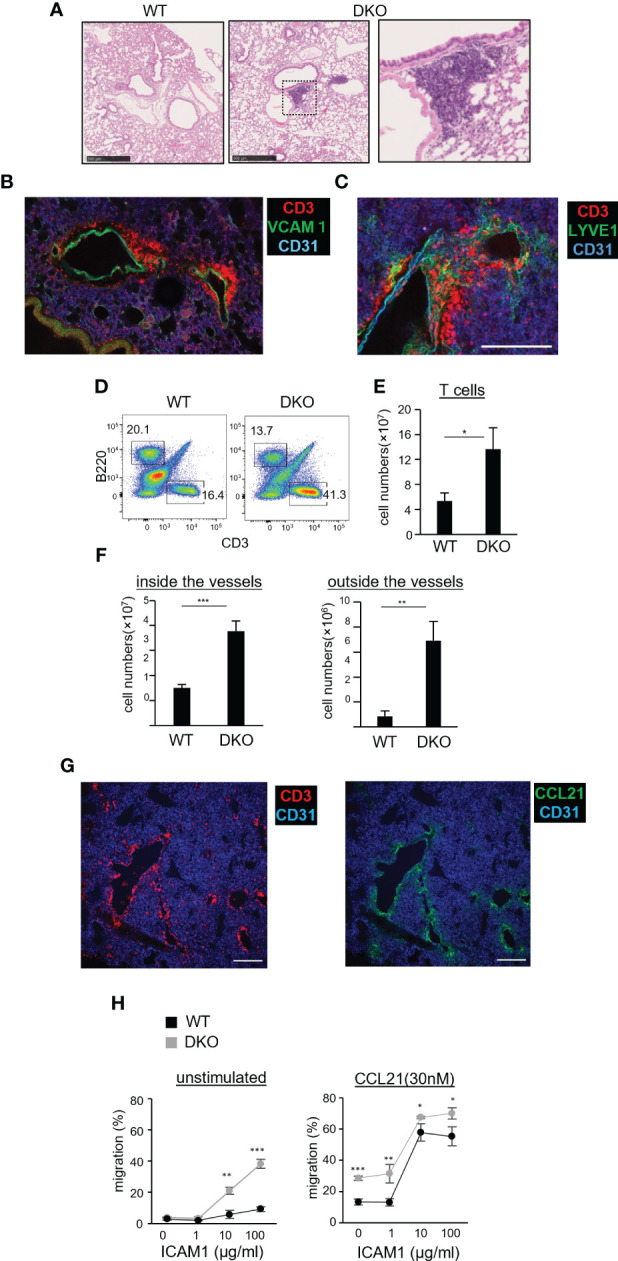
Naïve T-cell accumulation in the lung parenchyma of Rasa3- and Sipa1-deficient mice. **(A)** Representative images of tissue sections from the lung of WT (left image) and DKO mice (middle image) stained with hematoxylin and eosin **(H, E)**. The enlarged image (right image) shows an immune cell cluster in the perivascular area of DKO mice. Scale bar, 500 μm. **(B)** Three-dimensional reconstituted image of CD3 (red), CD31 (blue), and VCAM1 (green) in a vibratome-cut lung section from a DKO mouse. Scale bar, 10 μm. **(C)** Three-dimensional reconstituted image of CD3 (red), CD31 (blue), and LYVE1 (green) in a vibratome-cut lung section of DKO mice. Scale bar, 200 μm. **(D)** Representative flow cytometric profiles of CD3 and B220 in the lungs of WT and DKO mice using a lymphocyte gate. **(E)** The numbers of T cells (CD3^+^) in the lungs of the mice described in **(D)**. **(F)** Graph shows increased numbers of naïve T cells in both the intravascular space and LN parenchyma of DKO mice. Mice were intravenously injected with allophycocyanin-conjugated anti-CD3 antibody to label intravascular T cells. Five minutes later, their lungs were harvested. Cell suspensions from the harvested lungs were further stained with fluorescently labeled anti-TCRβ (FITC) and CD44 (PE) antibody. T cells in venules (CD3^+^TCR^+^) and those in LN parenchyma (CD3^−^TCR^+^) were analyzed by flow cytometry (n = 3). Naïve T cells were distinguished by low CD44 expression. **(G)** Images of CCL21 and T cells in the perivascular region of the lungs of DKO mice. Scale bar, 200 μm. **(H)** Migration toward CCL21 of WT T cells and DKO T cells on immobilized ICAM1 in the absence or absence (right panel) of CCL21 (30 nM) measured by transwell assay. The percentage of migrated cells (± SD) are shown. The statistical significance of the above data was calculated by Student’s t-test. ^*^P <.05, ^**^P <.01. ^***^P <.001.

The above results prompted us to examine the lungs of DKO mice aged 12 weeks and older. We consistently found clustered T cells in the lungs of these mice (**Figure 5A**). This phenotype was T-cell intrinsic, because mice reconstituted with bone marrow from DKO mice also showed this phenotype (**Figure S4A**). T cells accumulated near CD31^+^VCAM1^+^ venules in the lungs (**Figure 5B**), and closely associated with LYVE1^+^ lymphatic vessels (**Figure 5C**). The number of T cells in the lungs of DKO mice was increased by 2.5-fold compared to WT mice (**Figures 5D, E**). The majority of T cells in the lungs of DKO mice were naïve T cells (**Figures S4B, C**). To confirm whether the increased T cells in the lungs were located in the parenchyma or within blood vessels, we performed *in vivo* antibody labeling experiments to mark cells within blood vessels. While the number of labeled T cells was 2.5-fold higher in DKO mice than in WT mice, the number of non-labeled T cells was further increased 8-fold in DKO mice, indicating that T cells accumulated more in the lung parenchyma than within pulmonary vessels (**Figure 5F**). Collectively, these results indicated that in DKO mice, T cells accumulated in the perivascular areas associated with lymphatic vessels.

To gain insight into how T cells accumulated in pulmonary lymphatic vessels in DKO mice, we examined the expression of CCL21, a chemokine that plays a major role in attracting naïve T cells. We found that the perivascular areas exhibiting T-cell clustering showed abundant CCL21 expression (**Figure 5G**). We measured chemotactic responses of DKO T cells to CCL21 by transwell assays. Compared to WT T cells, DKO T cells exhibited high motility across membranes coated with ICAM1 (**Figure 5H**, left), and this motility was further enhanced in the presence of CCL21 (**Figure 5H**, right). The expression of CCR7 in DKO T cells was comparable to that of WT T cells (**Figure S4D**). Collectively, these data suggest that the enhanced chemotactic responses of DKO T cells cause them to accumulate in the perivascular lymphatics of the lung.

### Defective egress of DKO T cells from LNs

**Figure 6:**
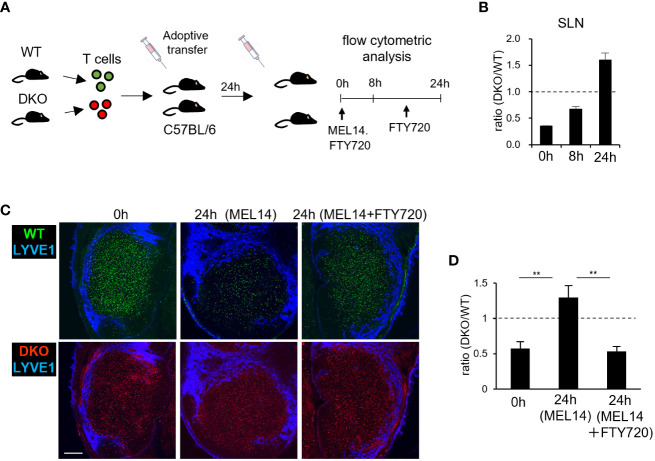
Impaired egress of Rasa3- and Sipa1-deficient T cells from LNs **(A)** Schematic diagram of LN egress assay. Equal numbers of labeled WT and DKO T cells were adoptively transferred to recipient mice. Twenty-four hours after transfer, anti-CD62L (MEL14) was intravenously injected to inhibit further entry. In some cases, FTY720 was intraperitoneally injected twice to block egress. At 0 hours, 8 hours, and 24 hours after injection, donor cells were analyzed by flow cytometry. **(B)** The bars indicate the mean ratio ± SD of DKO T cells to WT T cells in SLNs at 0 hours, 8 hours, and 24 hours. **(C)** Representative images of transferred WT T cells (green, upper) and DKO T cells (red, lower) in LN sections 0 hours (left) and 24 hours (center) after MEL14 treatment and 24 hours after treatment with both MEL14 and FTY720 (right). Lymphatic sinuses were visualized by anti-LYVE1 immunostaining (blue). Scale bar, 200 μm. **(D)** The bars indicate the mean density ratio ± SD of DKO T cells to Rasa3^fl/fl^ T cells in LN sections in the above conditions. The statistical significance of the above data was calculated by Student’s t-test. ^**^P <.01.

Johansen et al. reported that Rasa3-deficient T cells exhibited defective egress from LNs, though the underlying mechanism has yet to be determined (33). To investigate the egress of DKO T cells from LNs, fluorescently labeled WT and DKO T cells were adoptively transferred into WT recipients (**Figure 6A**). After 24 hours, the entry of transferred T cells into SLNs was blocked by injection with an anti-L-selectin antibody (MEL14). We assessed the numbers of transferred WT and DKO T cells in SLNs by flow cytometry. Before entry blocking, the ratio of DKO T cells to WT T cells in SLNs was 0.35 (**Figure 6B**). The ratio of DKO to WT T cells increased to 0.7 at 8 hours and to 1.5 at 24 hours post blocking, indicating that DKO T cells exited from SLNs at slower rates than WT T cells (**Figure 6B**). Confocal microscopic examination of fixed SLNs at 24 hours after transfer showed that both WT and DKO T cells were widely distributed in the paracortical areas, and the ratio of DKO to WT T cells was approximately 0.5 (**Figures 6C, D**). Twenty-four hours after MEL14 injection, the ratio of DKO to WT T cells increased to more than 1.0 (**Figures 6C, D**). Administration of FTY720, a S1P antagonist (40–43) maintained the ratio of DKO to WT T cells (**Figures 6C, D**), confirming S1P-dependent egress (6). These results indicate that the loss of Rasa3 and Sipa1 in T cells led to defective egress from LNs.

### Reverse migration of DKO T cells from lymphatic sinuses to LN parenchyma

**Figure 7:**
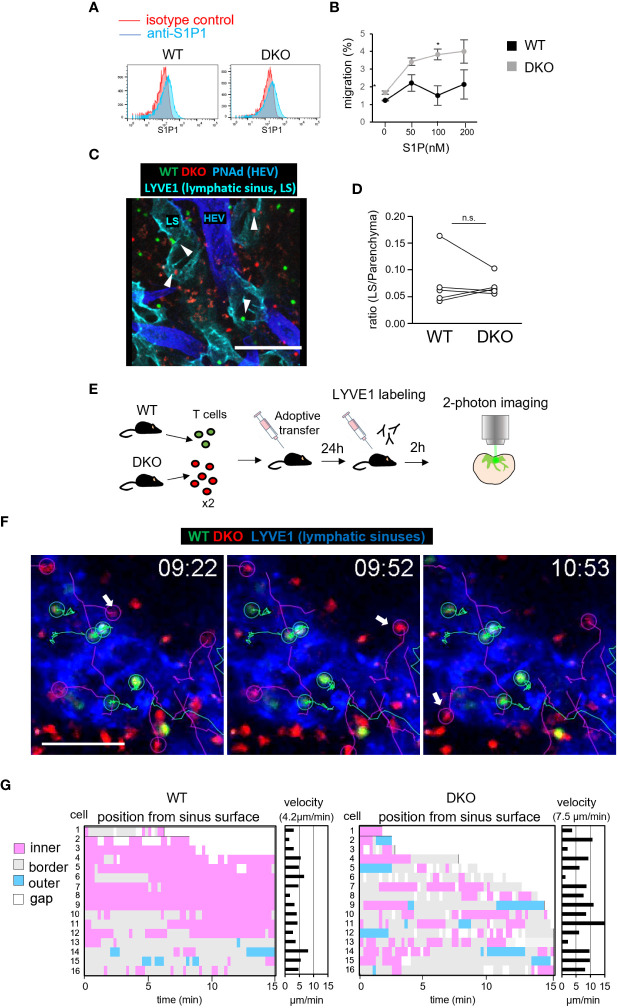
Rasa3- and Sipa1-deficient T cells in lymphatic vessels return to the T-cell zone. **(A)** The expression of S1P1 on T cells from WT and DKO mice measured by flow cytometry. T cells were stained with anti-S1P1 (blue) or isotype control (red). **(B)** The percentages of WT T cells (black circle) and DKO T cells (gray circles) migrating toward various concentrations (0–200 nM) of S1P, measured by transwell assay. **(C)** Representative images of donor WT T cells (green), donor DKO T cells (red), HEVs (blue), and lymphatic sinuses (cyan) in LN sections 4 hours after MEL14 treatment. White arrowheads represent donor cells in the lymphatic sinus. **(D)** The line graph shows the ratio of transferred WT and DKO T cells in lymphatic sinuses (LYVE1^+^) to those in LN parenchyma (5 LNs from 2 mice). **(E)** Schema of time-lapse imaging of WT T cells and DKO T cells in the medullary sinus region. The front footpads of mice that had been adoptively transferred with WT T cells and DKO T cells were injected with anti-LYVE1 antibody to visualize lymphatic sinuses. LNs were harvested and subjected to explant time-lapse imaging (15.2 seconds/frame) with 2-photon laser scanning microscopy. **(F)** Time-lapse 2D projection images of WT T cells (green), DKO T cells (red), and lymphatic sinuses (blue) with cell trajectories (timepoints mm:ss) in the medullary regions. A TrackMate plug-in of ImageJ was used to track cells. Arrows indicate DKO T cells which migrated from lymphatic sinuses to the LN parenchyma. Scale bar, 50 μm. **(G)** The positions of individual T cells from the surfaces of LYVE1^+^ lymphatic sinuses was measured using the surface distance option of Imaris software. Bars represent distances of individual T cells to the LYVE^+^ surface, categorized as follows: outer (light blue), >2.5 μm from the surface; border (gray), within 2.5 μm from the surface; inner (pink), >2.5 μm from the surface; and gap of a track (white). X axis represents time (min) The average velocities of the individual WT and DKO T cells are also shown in the right panel. The number in parentheses shows the average velocity of the T-cell population. The statistical significance of the above data was calculated by Student’s t-test. ^*^P <.05, n.s: not significant.

To determine if the defective egress of DKO T cells might be due to an impaired chemotactic response to S1P, we measured S1P1, a receptor of S1P expression by flow cytometry. S1P1 expression was comparable in WT and DKO T cells (**Figure 7A**). Furthermore, *in vitro* chemotactic responses to S1P were enhanced in DKO T cells relative to WT T cells, ruling out the possibility that a defective S1P response impairs the egress of DKO T cells (**Figure 7B**). To identify the mechanism underlying the defective egress of DKO T cells, we measured the migration efficiency of DKO T cells from the LN parenchyma to lymphatic sinuses. For this purpose, we adoptively transferred WT and DKO T cells to WT recipients, and compared the ratio of the number of T cells in lymphatic sinuses to that of T cells in the LN parenchyma 4 hours after entry blocking with MEL14 (**Figures 7C, D**). The ratio was comparable between WT and DKO T cells (**Figures 7C, D**), suggesting normal entry of DKO T cells into the lymphatic sinuses from the LN parenchyma.

The intranodal motility of lymphocytes may affect the efficiency with which they encounter lymphatic sinuses (44). Therefore, we measured the migration velocity of WT and DKO T cells within LN tissues by time-lapse imaging using 2-photon microscopy (3). WT T cells and DKO T cells exhibited intranodal velocities of 11 μm/min and 10 μm/min, respectively, with comparable meandering indexes (**Figure S5**; **Video S1**). We considered it unlikely that this small difference would cause the severe defect in the egress of DKO T cells. To examine the cellular dynamics of DKO T cells in the process of LN egress, we adoptively transferred WT and DKO T cells into WT mice and measured T-cell migration in the medullary sinus areas of explanted LNs by 2-photon microscope (**Figure 7E**). To visualize lymphatic sinuses, recipient mice were subcutaneously injected with fluorescently labeled LYVE1-specific antibody 2 hours before live imaging. We tracked T cells in LYVE1^+^ lymphatic sinuses and measured the distance of T cells from the surface of lymphatic sinuses (**Figures 7F, G**). Among measured T cells, Most WT T cells migrated slowly and remained in the lymphatic sinuses (**Figures 7F, G**; **Video S2**). In contrast, DKO T cells in the lymphatic sinuses were more mobile and frequently returned back to the LYVE1^–^ LN parenchyma (WT, 1 of 16 cells, DKO 5 of 16 cells among we measured, **Figure 7G**). DKO T cells consistently migrated faster than WT T cells in lymphatic sinuses (average velocity: WT T cells, 4.2 μm/min; DKO T cells, 7.1 μm/min; *P* = 0.017). These results strongly suggest that the loss of Rasa3 and Sipa1 in T cells augmented reverse migration from lymphatic sinuses to the LN parenchyma, and thereby compromised egress from LNs.

## Discussion

Here we showed that Rasa3 and Sipa1 play a critical role in T-cell homeostasis involving Rap1 inactivation. Inactivation of both Rasa3 and Sipa1 in T cells caused greater activation of Rap1 than inactivation of either Rasa3 or Sipa1, which correlated with increased adhesiveness of LFA1. Furthermore, T cells deficient in Rasa3 and Sipa1 exhibited greater motility and chemotactic responses to CCL21 and S1P than WT T cells. These properties caused T-cell lymphopenia in the periphery of DKO mice due to impaired T-cell recirculation through the vascular and lymphatic systems. Specifically, DKO T cells were entrapped in the ICAM-rich pulmonary vascular bed by LFA1 in a process that required Rap1 and talin1, resulting in an inadequate supply of T cells into peripheral lymphoid tissues. Furthermore, DKO T cells inefficiently exited from LNs due to accelerated reverse migration from lymphatic sinuses to the LN parenchyma. Collectively, our study identified the critical trafficking steps that maintain efficient T-cell recirculation *via* inactivation of Rap1.

Recirculating T cells pass through the lungs before they travel to the LNs and spleen. A mathematical model predicted that naïve T cells spend only a minute in the lungs (45). Thus, this short time is critical for efficient T-cell recirculation. The finding that DKO T cells were entrapped in the lungs for substantially longer times emphasizes the importance of strict regulation of integrin activity in their passage through ICAM-rich pulmonary beds. We showed that blocking LFA1 or inactivating Rap1 and talin1 rescued the entrapment of DKO T cells. The contribution of LFA1/ICAM interaction to lung entrapment was previously reported in activated effector T cells (38). Lee et al. reported that platelets from mice bearing a hypomorphic *Rasa3* mutant were entrapped in the spleen and liver, and subsequently they were engulfed by macrophages, presumably by talin1-dependent activation of integrin αIIbβ3, thus causing thrombocytopenia (46). The DKO T cells entrapped in the pulmonary capillaries probably transmigrate into the lung parenchyma, leading to accumulation in lung perivascular lymphatics that exclusively express CCL21. Rap1 interacts with talin1 directly or through RIAM (47–49), and recruits talin1 to the cytoplasmic tail of integrin β-subunits, resulting in conformational and affinity changes in integrins (10, 11). Our study indicates that the Rap1-talin axis that controls integrins in naïve T cells is basally suppressed by Rasa3 and Sipa1, and that this is critical for these cells’ passage in the pulmonary vascular network.

Several studies showed that LFA1 mutants with increased affinity to ligands inhibited lymphocyte extravasation through HEVs (50, 51). Semmrich et al. (50) generated knock-in mice (LFA1^d/d^ mice) expressing constitutively active LFA1 by disrupting an inhibitory αβ salt bridge in the LFA1 cytoplasmic tail. T cells from LFA1^d/d^ mice showed migration defects due to defective de-adhesion to ICAM1, resulting in impaired transendothelial migration with a decreased number of lymphocytes in LNs. Similar results were also reported in knock-in mice expressing constitutively active LFA1 (α_L_-I306A mice) (50, 51). By contrast, the absence of Rasa3 and Sipa1 did not appear to affect the ability of naïve T cells to enter the LN parenchyma *via* HEVs, despite spontaneous integrin activation due to increased activation of Rap1. The reason for this difference is that unlike constitutively active LFA1 mutants, Rap1 activation and ICAM1 binding together shift the conformation equilibrium of LFA1 toward high-affinity states, and thus some LFA1 molecules temporarily exhibited high-affinity ligand binding in DKO T cells (2). In addition, Rap1 activation or overexpression of constitutively active Rap1 was found to induce cell polarity and enhance cell motility on ICAM1 (9), as also shown in this study. The difference in the process underlying LFA1 activation associated with cell motility likely causes the discrepant outcomes (52, 53).

DKO T cells exhibited delayed egress from LNs, as reported with Rasa3 deficiency (33). This was not due to impairment in S1P responses, or in the access and entry into lymphatic sinuses, unlike S1P1 knockout T cells (6). By using live imaging of DKO T cells in explanted LNs, we showed that DKO T cells entered lymphatic sinuses but frequently moved back to the LN parenchyma, suggesting enhanced reverse migration from lymphatic sinus to LN parenchyma as the underlying mechanism of delayed egress. Of note, the reverse migration of T cells in the medullary region was reported as non-negligible physiological process (7, 34, 44). Alternatively, DKO T cells may exhibit enhanced adhesiveness of LFA1 and/or enhanced chemotactic response to CCL21, thereby causing retention of DKO T cells within lymph nodes instead of the reverse migration, since previous studies showed that LFA1-mediated adhesion and CCR7-mediated chemotaxis acted as a retention factor within LNs to inhibit egress (34, 54). However, our results demonstrate that DKO T cells exhibited normal efficiencies of LN entry *via* HEV, the process inhibited by high-affinity LFA1 *via* enhanced stable adhesion. DKO T cells also did not considerably alter interstitial migration profiles or exhibit stable arrest within T-cell zones. Moreover, histological examinations of LNs revealed that DKO T cells was not retained in the LN parenchyma and entered lymphatic sinuses as efficiently as WT cells. In support of these observations, DKO T cells showed enhanced chemotaxis to both S1P and CCL21, but not stable adhesion, even in the presence of ICAM1 *in vitro*. Based on these results, the reverse migration to the LN parenchyma provides a plausible explanation for the defective egress of DKO T cells, although we could not formally exclude the possibility that the delayed egress of T cells is due to enhanced adhesiveness of LFA1 and CCR7-mediated chemotaxis.

It is currently unclear how Rasa3 and Sipa1 are regulated in T cells. The PIP3 binding domain of Rasa3 is reported to be important for Rasa3 intracellular localization (33). We previously reported that Sema3e and its receptor PlexinD1 induced Rap1 inactivation and altered both thymocyte migration in the thymus and immune synapse formation (36). Considering the importance of Rap1 for immune surveillance and antigen responses, there may exist physiological regulators of Rasa3 and Sipa1 that inactivate Rap1. Our study provides important clues to the search for such molecules.

## Data availability statement

The original contributions presented in the study are included in the article/**Supplementary Material**. Further inquiries can be directed to the corresponding authors.

## Ethics statement

The animal study was reviewed and approved by The Animal Care and Use Committee of Kansai Medical University.

## Author contributions

SH, YU, and TK conceived and designed the experiments. SH and YU carried out the experiments and data analysis. SH, YU, and YK generated the single, double, triple, knockout mice. SH, YU, TK, NK, YI, YK, and MN interpreted the data. YU, TK, and SH wrote and edited the manuscript. All authors contributed to the article and approved the submitted version.
