# Supplementary material for: The critical role of Rap1-GAPs Rasa3 and Sipa1 in T cells for pulmonary transit and egress from the lymph nodes
Source: Front Immunol. 2023 Jul 20;14:1234747. doi: 10.3389/fimmu.2023.1234747 (PMC10399222; doi:10.3389/fimmu.2023.1234747)
Supplement: Supplementary file 1 [file DataSheet_1.docx]

**Supplemental Text**

**Video S1 Interstitial migration of DKO T cells within LNs.**

WT (green) and DKO T cells (red) with tracks within LN slices measured by 2-photon laser scanning microscope. Blue: autofluorescence, 15 sec/frame. 1 box, 25 μm

**Video S2 Migration of DKO T cells in medullary sinus regions of LNs.**

Time lapse 2D projection images of WT T cells (green), DKO T cells (red), and lymphatic sinus (blue) in the medullary sinus regions with track. Scale bar, 50 μm. 15.2 sec/frame.
