# Supplementary material for: The critical role of Rap1-GAPs Rasa3 and Sipa1 in T cells for pulmonary transit and egress from the lymph nodes
Source: Front Immunol. 2023 Jul 20;14:1234747. doi: 10.3389/fimmu.2023.1234747 (PMC10399222; doi:10.3389/fimmu.2023.1234747)
Supplement: Supplementary file 2 [file DataSheet_2.pdf]

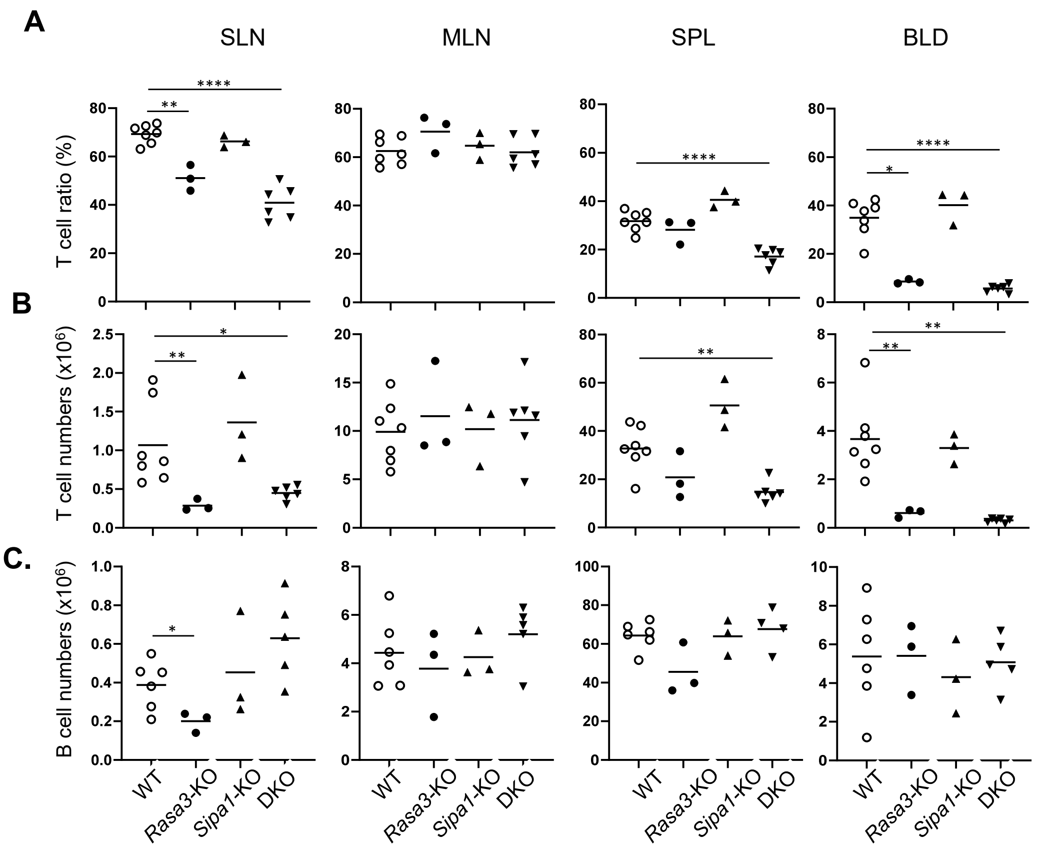

Figure S1. T-cell lymphopenia in Rasa3- and Sipa1-deficient mice.
(**A**) The percentages of $CD3^+$ T cells in the superficial and mesenteric LNs (SLN, MLN), spleen (SPL), and blood (BLD) of WT, Rasa3-KO, Sipa1-KO and DKO T cells measured by flow cytometry. Bars indicate means. (**B**) The numbers of $CD3^+$ T cells in the SLN, MLN, SPL, and BLD of the above mice. (**C**) The numbers of $B220^+$ B cells in the SLN, MLN, SPL BLD of the above mice. The data of WT and DKO mice are the same as in Figure 2.

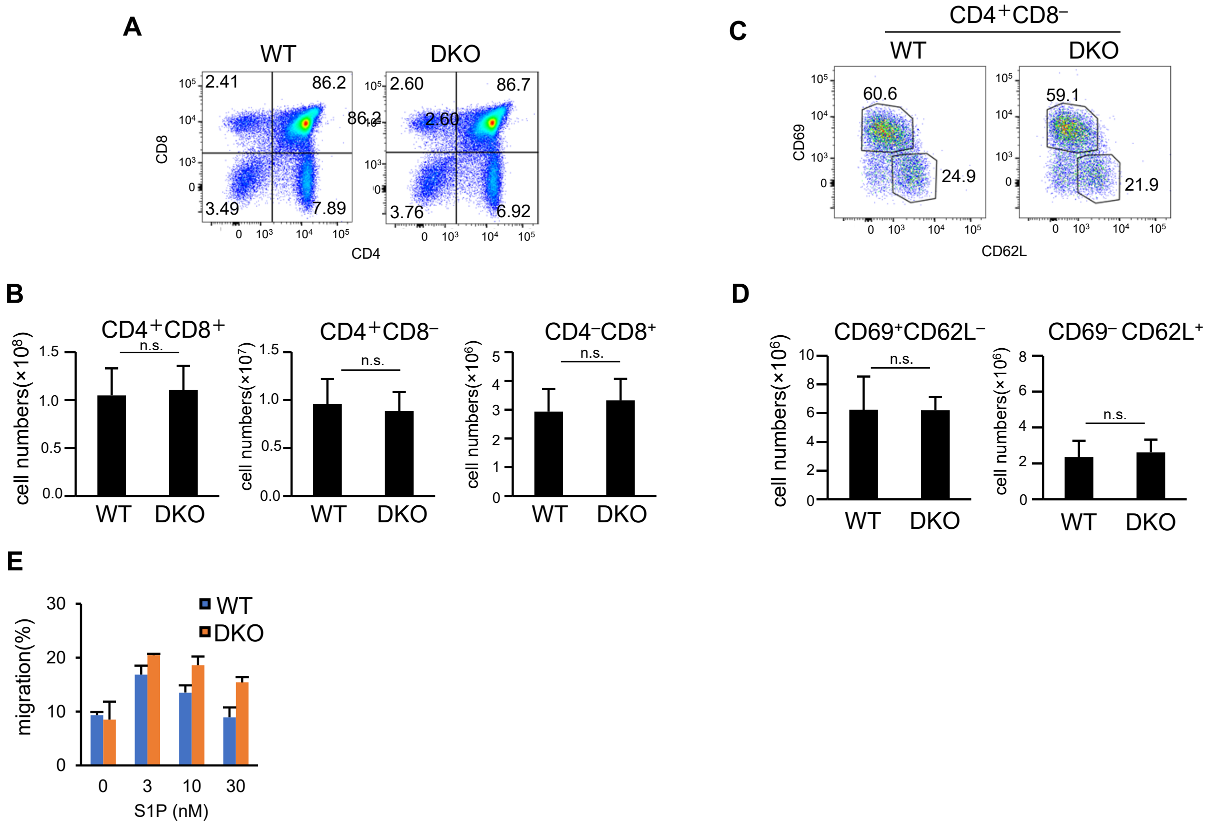

Figure S2. Normal thymocyte development in Rasa3- and Sipa1-deficient mice.
(**A**) CD4 and CD8 profiles of thymocytes in WT and DKO mice. (**B**) The numbers ± SD of CD4$^+$CD8$^+$, CD4$^+$SP, and CD8$^+$SP cells in (A). n.s: not significant (n = 3). (**C**) DKO thymocyte egress was normal. CD69 and CD62L expression in CD4 SP thymocytes in the above mice. (**D**) The numbers ± SD of CD62L$^-$CD69$^+$ and CD62L$^+$CD69$^-$ CD4P cells (n = 3). (**E**) Migration toward S1P of CD62L$^+$CD69$^-$ CD4SP cells in WT (blue) and DKO (red) thymocytes measured by transwell assay. The percentages (± SD) of migrated CD62L$^+$CD69$^-$ CD4SP cells are shown. Triplicates from a representative of two independent experiments. The statistical significance of the above data was calculated by Student's t-test. $^*P < .05$, $^{**}P < .01$. $^{***}P < .001$, n.s.: not significant.

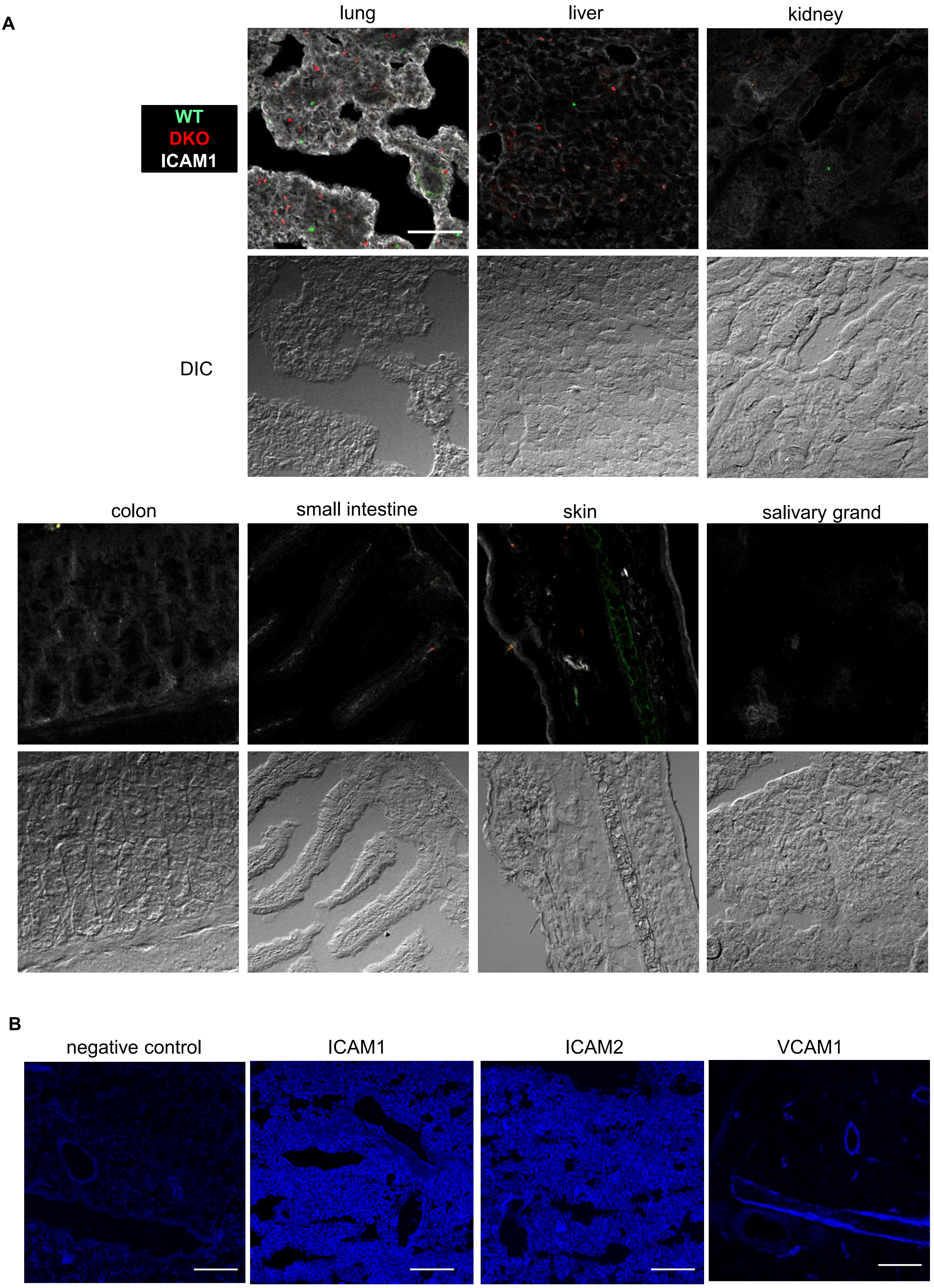
A
lung
liver
kidney
WT
DKO
ICAM1
DIC
colon
small intestine
skin
salivary grand
B
negative control
ICAM1
ICAM2
VCAM1

Figure S3. Rasa3- and Sipa1-deficient T cells accumulate in lungs and their numbers correlate with high ICAM1 expression.

(**A**) Outside of non-hematopoietic tissues, DKO T cells predominantly accumulated in the lungs. Confocal images show WT T cells (green), DKO T cells (red), and ICAM1 (white) in non-hematopoietic tissue sections from lung, liver, kidney, colon, small intestine, skin, and salivary grand. Differential interference contrast microscopy images of the above tissues are shown in the lower panel. Scale bar, 100 μm. (**B**) Representative confocal images of ICAM1, ICAM2, and VCAM1 expression in the lungs. A negative staining control is also shown. Scale bar, 100 μm.

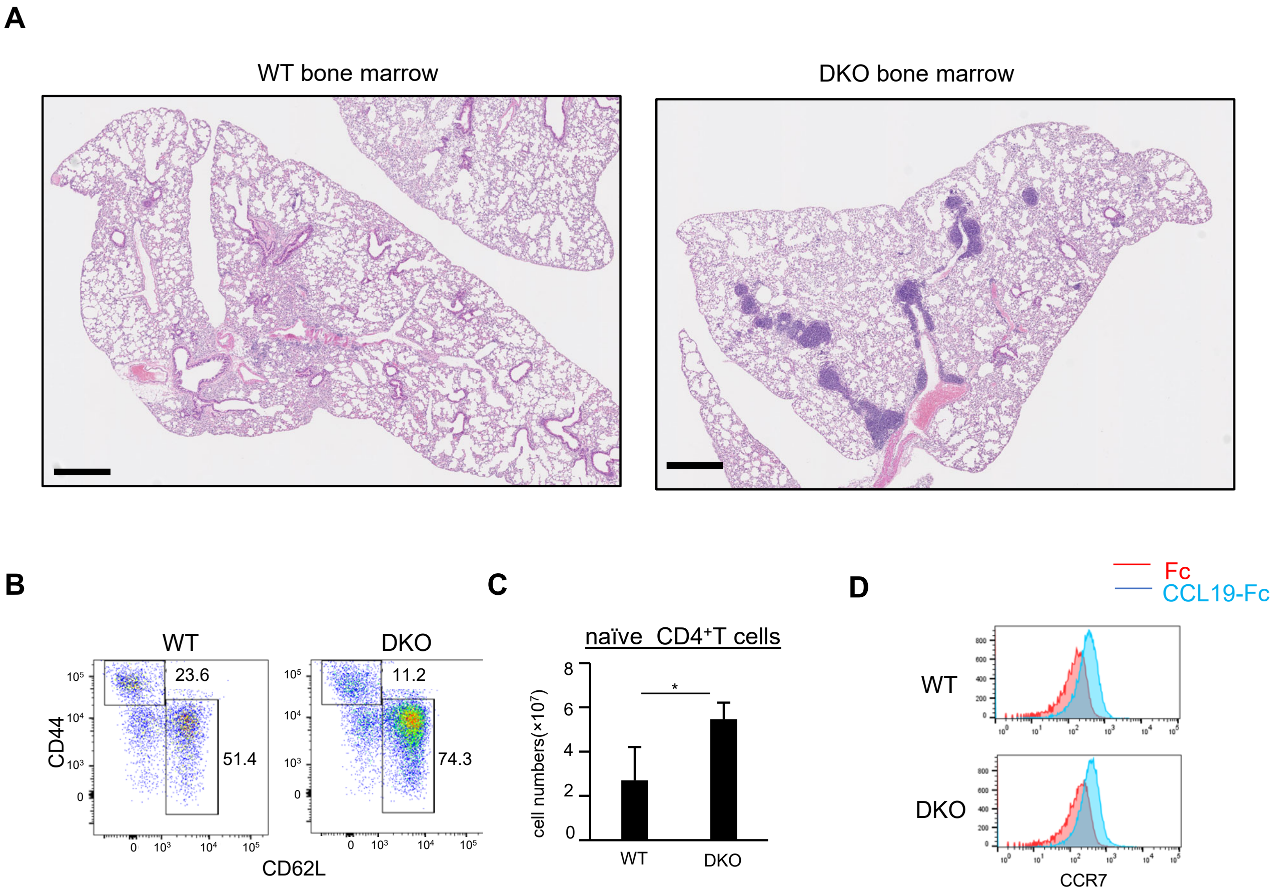

Figure S4. T cells accumulate in lymphatics in the perivascular space in the lungs of Rasa3- and Sipa1-deficient mice.

(**A**) Representative images of H&E-stained lung sections from mice reconstituted with WT bone marrow (BM) (left) or DKO BM (right) (n = 3, 4–6 months). (**B**) Expression profiles of CD62L and CD44 in CD4$^+$ T cells in the lungs of the mice described above in (D). Square gates for CD44^{low}CD62L^{high} and CD44^{high}CD62L^{low} represent naive and effector T cells, respectively. (**C**) The numbers of naive CD4$^+$ T cells (CD62L^{high}CD44^{low}) in mouse lungs. (**D**) Expression of CCR7 on T cells from *Rasa3*$^{fl/fl}$ and *Cd4-cre Rasa3*$^{fl/fl}$*Sipa1*$^{-/-}$ mice measured by flow cytometry. T cells were stained with CCL19-Fc (blue) or Fc control (red) and visualized by anti-human-immunoglobulin antibody conjugated with FITC.

Figure S4

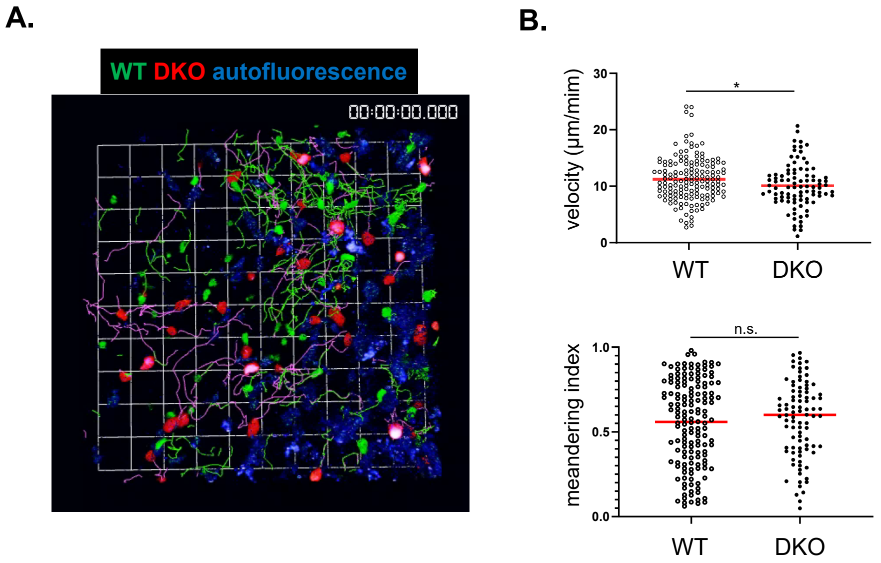

Figure S5. Interstitial migration of DKO T cells within LNs.
(**A**) Migration tracks of WT (green) and DKO T cells (red) within LN slices measured by 2-photon laser scanning microscope. (**B**) The mean migration velocities and meandering indexes of individual WT and DKO T cells within a LN slice. The red bars show means. Statistical significance was calculated by the Mann-Whitney U test. *$P < 0.05$, n.s.: not significant.
